# Supplementary material for: Emergency Preparedness for the COVID-19 Pandemic: Social Determinants Predicting the Community Pharmacists’ Preparedness and Perceived Response in Malaysia
Source: Int J Environ Res Public Health. 2022 Jul 19;19(14):8762. doi: 10.3390/ijerph19148762 (PMC9315557; doi:10.3390/ijerph19148762)
Supplement: Supplementary file 1 [file ijerph-19-08762-s001.zip › ijerph-1709894-supplementary.pdf]

Preparedness towards COVID-19

1. I have all the information related to the needs of my community regarding COVID-19.
   Yes ☐ No ☐
2. I am aware of the challenges that I need to face from my community regarding COVID-19.
   Yes ☐ No ☐
3. I know where to get the resources/materials needed for my community in this COVID-19 situation.
   Yes ☐ No ☐
4. I am aware of the programs regarding COVID-19 preparedness and management that are offered by Ministry of Health.
   Yes ☐ No ☐
5. I read journal articles related to COVID-19 preparedness.
   Yes ☐ No ☐
6. I know whom to contact (chain of command) in disastrous situation in my community.
   Yes ☐ No ☐
7. I find that the research information on COVID-19 management is easily accessible from my pharmacy setting.
   Yes ☐ No ☐
8. I have participated in educational activities dealing with COVID-19 preparedness recently (ex: continuing education, webinars, or conferences)
   Yes ☐ No ☐
9. I agree that history should be taken on whether the customers has resided in or travelled to a country.
   Yes ☐ No ☐
10. In case of emergency, I know how to use personal protective equipment.
    Yes ☐ No ☐
11. In case of emergency, I know how to execute decontamination procedures within the pharmacy.
    Yes ☐ No ☐
12. I am familiar with accepted triage principles used in emergency situations.
    Yes ☐ No ☐
13. In a case of emergency, I know how to perform isolation procedures to minimise the risks of community exposure.
    Yes ☐ No ☐
14. I consider myself prepared for the management of COVID-19 outbreak.
    Yes ☐ No ☐
15. I am ready for peer evaluation of my skills on preparedness to COVID-19.
    Yes ☐ No ☐

Perceived response efficacy towards COVID-19

16. I am confident in providing patient education on COVID-19.
    Yes ☐ No ☐
17. I can identify the signs and symptoms of COVID-19.
    Yes ☐ No ☐

18. I am confident that I can perform my duties in COVID-19.
    Yes ☐ No ☐
19. I can respond as a direct-care provider or first responder in COVID-19.
    Yes ☐ No ☐
20. I can manage COVID-19 patients independently without any supervision.
    Yes ☐ No ☐
21. I can respond to patients with worsen symptoms and reactions of COVID-19.
    Yes ☐ No ☐
22. There are enough medications needed to manage the COVID -19 emergency.
    Yes ☐ No ☐
23. There are enough PPE needed to manage the COVID -19 emergency.
    Yes ☐ No ☐
24. I personally have received clients needing help with Covid-19 issues.
    Yes ☐ No ☐
25. I can identify possible indicators of mass exposure evidenced by a clustering of patients with similar symptoms
    Yes ☐ No ☐
26. I am ready for peer evaluation of my skills on responsiveness to COVID-19.
    Yes ☐ No ☐
